# Treatment of depressive disorders in primary care - protocol of a multiple treatment systematic review of randomized controlled trials

**DOI:** 10.1186/1471-2296-12-127

**Published:** 2011-11-15

**Authors:** Klaus Linde, Isabelle Schumann, Karin Meissner, Susanne Jamil, Levente Kriston, Gerta Rücker, Gerd Antes, Antonius Schneider

**Affiliations:** 1 Institute of General Practice, Technische Universität München, Orleansstr. 47, D-81667 München, Germany; 2 University Medical Center Hamburg-Eppendorf, Department of Medical Psychology, Martinistr. 52, D-20246 Hamburg, Germany; 3 Institute of Medical Psychology, Ludwig-Maximilians-University Munich, Goethestr. 31, D-80336 München, Germany; 4 Department of Medical Biometry and Statistics, University Medical Center Freiburg, Stefan-Meier-Str. 26, D-79104 Freiburg, Germany

## Abstract

**Background:**

Several systematic reviews have summarized the evidence for specific treatments of primary care patients suffering from depression. However, it is not possible to answer the question how the available treatment options compare with each other as review methods differ. We aim to systematically review and compare the available evidence for the effectiveness of pharmacological, psychological, and combined treatments for patients with depressive disorders in primary care.

**Methods/Design:**

To be included, studies have to be randomized trials comparing antidepressant medication (tricyclic antidepressants, selective serotonin reuptake inhibitors (SSRIs), hypericum extracts, other agents) and/or psychological therapies (e.g. interpersonal psychotherapy, cognitive therapy, behavioural therapy, short dynamically-oriented psychotherapy) with another active therapy, placebo or sham intervention, routine care or no treatment in primary care patients in the acute phase of a depressive episode. Main outcome measure is response after completion of acute phase treatment. Eligible studies will be identified from available systematic reviews, from searches in electronic databases (Medline, Embase and Central), trial registers, and citation tracking. Two reviewers will independently extract study data and assess the risk of bias using the Cochrane Collaboration's corresponding tool. Meta-analyses (random effects model, inverse variance weighting) will be performed for direct comparisons of single interventions and for groups of similar interventions (e.g. SSRIs vs. tricyclics) and defined time-windows (up to 3 months and above). If possible, a global analysis of the relative effectiveness of treatments will be estimated from all available direct and indirect evidence that is present in a network of treatments and comparisons.

**Discussion:**

Practitioners do not only want to know whether there is evidence that a specific treatment is more effective than placebo, but also how the treatment options compare to each other. Therefore, we believe that a multiple treatment systematic review of primary-care based randomized controlled trials on the most important therapies against depression is timely.

## Background

Epidemiological studies indicate that depressive disorders are highly prevalent in the general population worldwide [1]. Most cases are seen and managed in primary care, and only a small proportion of these are referred to specialty care [2]. A number of studies suggest that primary care patients with depressive disorders are less severely depressed [3], experience a milder course of illness [4], have a distinct symptom profile with more complaints of fatigue and somatic symptoms [5], and are more likely to have accompanying physical complaints [6] than patients referred to specialty mental health care.

The cornerstones of antidepressant treatment are pharmacotherapy and psychological interventions [7]. However, while the vast majority of patients with depression are dealt with in primary care, most of the research findings upon which decisions are made have involved secondary care patients. It is not fully clear whether the findings from trials in specialty settings can be generalized to primary care. Meta-analyses restricted to primary care patients have been performed for SSRIs and tricyclics compared to placebo [8,9], SSRIs compared to tricyclics [10], and psychological interventions [11,12]. They concluded that these treatments are effective in primary care settings. In some countries a relevant proportion of primary care patients with depressive symptoms is treated with hypericum extracts [13]. The co-morbidity and symptom pattern of primary care patients described in recent studies [14,15] fits well to the traditional indications of hypericum extracts (psycho-vegetative disorders, depressive disorders, anxiety and/or nervous agitation) [16]. Systematic reviews of hypericum extracts include a considerable number of randomized trials in primary care patients [17,18]. However, in these reviews the results of trials in primary and secondary care settings were pooled and not analyzed separately. Systematic reviews on music therapy, acupuncture, exercise, relaxation, and family therapy for treating depression published in the Cochrane Database of Systematic Reviews include only few or no trials conducted in primary care settings [19-23].

The systematic reviews and meta-analyses cited above [8-12,17,18] summarize the majority of the available randomized trials of depression treatments in primary care. However, it is not possible to answer the question how the available treatment options compare with each other (i.e., whether some treatments are superior to others in primary care). Traditional meta-analyses are restricted to the direct comparison of two interventions by pooling data only from trials with similar treatment arms. By consequence, they allow no decision about the relative effectiveness of two treatments, if they have not yet been directly compared in at least one randomized controlled trial (RCT). However, in case of insufficient or missing direct comparisons of available interventions the utility of indirect evidence may be considered. For example, RCTs of treatment A vs. placebo and treatment B vs. placebo would provide indirect estimates on the comparative effectiveness of A vs. B through the common reference placebo. The inclusion of more interventions would result in more complex networks and involve more complex indirect comparisons.

Network (or multiple/mixed treatment) meta-analyses are an enhancement of the traditional meta-analysis methodology to more than two interventions [24]. They estimate the comparative effectiveness between two treatments based on all available direct and indirect evidence that is available in a network of treatments and comparisons. Besides augmenting validity of comparisons between available treatments through including indirect evidence, network meta-analyses allow for a formal assessment of evidence inconsistencies. Not least, they suggest a ranking of interventions according to their relative effectiveness, which may be of high relevance for clinical decision making. Network meta-analyses have been performed, for example, to compare newer antidepressant agents [25,26]. However, most trials included in these research syntheses were not performed in the primary care setting. This is of importance as patients in primary care might differ from those in secondary care.

For results of network meta-analyses to be valid three important pre-conditions should be met [27]: 1) the findings of each of the meta-analyses of the direct comparisons should be homogeneous (not suggesting that trials investigated slightly different questions); 2) for the indirect comparisons to be valid, patients included in the separate subgroups of trials need to be sufficiently similar; and 3) if both direct and indirect comparisons are available the pooled estimates for these need to be consistent. It might be that these pre-conditions will not be met in case of the primary care trials of various treatments for depression.

### Objectives

We will systematically review all randomized trials investigating treatments for depression performed in primary care settings. As we will use the same review methods across all treatments, a comparison of the evidence regarding effectiveness, feasibility, and safety will be possible. If adequate, we aim to perform a formal multiple treatment meta-analysis.

## Methods/Design

### Criteria for selecting studies for this review

#### Type of study

Inclusion will be restricted to trials in which allocation of patients to groups was explicitly randomized. Trials in which a clearly inadequate method or a pseudo-random method was used (e.g. alternation) will be excluded. Trials with a post-randomization period of less than 4 weeks will be excluded.

#### Types of participants

To be included studies must have recruited adults (18 years or older). Studies with a majority (more than 50%) of participants under 18 years will be excluded. Patients must have been recruited from a primary care setting (primary care clinics, private practices of general practitioners, internists or other non-psychiatrists providing primary care in the respective country). Trials in mixed settings (for example, some patients recruited by general practitioners, some by psychiatrists in private practice) will be excluded unless subgroup data on primary care patients are available. Included patients had to suffer from prevalent or incident unipolar depressive disorder. Studies using ICD 10 (International Classification of Disease - WHO, tenth revision) for operationalizing diagnoses will be included if patients are classified under the categories F32.×/F33.× or F34.1, studies using DSM IV (Diagnostic and Statistical Manual - American Psychiatric Association) will be included if patients are classified under the categories 296.2×/296.3× or 300.4. As older or pragmatic studies in primary care often do not use such classification we will also include studies in which the provider considered patients to suffer from depression without applying a formal classification system if the diagnosis is assessed to be valid by the reviewers. Studies focussing exclusively on depressive symptoms in a specific group of patients suffering primarily from another condition (e.g., depressive symptoms in patients with cardiovascular disease, Alzheimer disease, or postpartum depression) will be excluded whereas studies including depressive primary care patients which also suffer from a variety of other conditions will be included. Studies in mixed patient populations (e.g. some with depression and some with cyclothymia) will be included only if findings for the subgroup of patients with depression are available separately.

#### Types of interventions

Based on preliminary searches we have decided to focus on interventions which are widespread and are included in recent guidelines for outpatient care [7,28]: tricyclic and tetracyclic antidepressants, selective serotonine reuptake inhibitors, monoamino-oxidase inhibitors, newer agents such as venlafaxine or mirtazepine, hypericum extracts, psychological and psychosocial therapies (interpersonal psychotherapy, cognitive therapy, behavioural therapy, short dynamically-oriented psychotherapy, counselling, etc.) or a combination of pharmacological and psychological therapy. If screening searches identify a relevant number of primary care trials on other interventions the inclusion criteria will be adapted. We will not include trials which investigate the management of treatment (for example interprofessional case-management) instead of the depression therapy as in such trials typically allow a mixture of different treatment options both in intervention and control groups.

#### Types of comparators

Trials will be included if they compare one of the interventions listed above to at least one of the following options: a) directly versus one of the other interventions listed under "Interventions"; b) versus placebo, sham or attention control procedures; c) versus usual care; d) versus waiting list or no treatment.

#### Types of outcome measures

To be included trials have to report results on at least one of the following outcomes: response to treatment, remission, mean score on a depression scale (post-treatment or change from baseline), quality of life, patient global assessment, number of patients with adverse or side effects, number of patients discontinuing treatment or dropping out from the study (for any reasons and/or lack of improvement and/or adverse effects).

### Search methods for identification of studies

In a first step a basic collection of studies will be compiled from existing systematic reviews restricted to randomized trials in primary care of tricyclic antidepressants and SSRIs [8-10], psychological therapies [11,12], as well as from a Cochrane review on hypericum extracts [17,18]. In order to identify further potentially relevant treatment interventions we will screen meta-analyses of other treatments for trials performed in primary care. Furthermore, we will screen the references yielded by a PubMed search for randomized trials in primary care patients with depressive disorders. We then will perform database searches without language restrictions in CENTRAL, MEDLINE and EMBASE. The search strategy will be developed in consultation with an information specialist and tested using the trials identified for the basic collection. All databases will be searched from 1980 onwards (older trials will not be searched systematically due to different diagnostic classifications and concerns regarding study quality) using both standard vocabulary (e.g. MeSH) and keywords. For searches a disease-component will be combined (AND) with a setting-component and a design-component. RCTs (design-component) will be identified using the Cochrane Highly Sensitive Search Strategy for identifying randomized controlled trials.

We will search trial registers (Clinicaltrials.gov, International Clinical Trials Registry Platform (ICTRP), German Clinical Trial Register) to identify ongoing and unpublished studies. Furthermore, we will examine reference lists of identified studies.

### Study selection process

Following a screening of titles and abstracts by one reviewer to exclude clearly irrelevant papers, the decision to include or exclude a potentially eligible study will be based on the review of full texts independently by at least two reviewers using a structured form with the selection criteria listed above. Disagreements between reviewers will be documented and resolved by discussion. If consensus cannot be achieved between the original reviewers, an additional reviewer will be asked to make a final decision.

### Data extraction

Primary study characteristics and results will be extracted by at least two independent reviewers using a pre-tested form. Extraction will be made in a manner similar to the Cochrane review of hypericum extracts of depression [18]. In particular, we will document diagnoses and main inclusion criteria, age, gender, duration of episodes, baseline depression scores, country of origin, number and type of study centres, numbers of patients who were randomized and analyzed and who completed protocols, the number and reasons for drop-outs and withdrawals, numbers of patients reporting adverse effects, and the number and type of adverse effects that were reported. We will extract numbers of patients who were classified as responders based on score improvements on the Hamilton Rating Scale for Depression (HAMD; first preference), the Montgomery-Asberg Depression Rating Scale (MADRS; second preference), the Clinical Global Impression Index (CGI; subscale global improvement rating as at least "much improved"; third preference), or any other clinical response measurement. We will try to obtain missing information from authors/sponsors. Means and standard deviations for observer-rated (e.g. HAMD, Montgomery-Asberg Depressions Rating Scale) or patient-rated scales (e.g. Depression Scale von Zerssen) will be documented, too. For studies which do not provide any response data the proportion of responders will be estimated from metric variables [29]. Extraction of studies included in existing meta-analyses will be cross-checked with the data used for effect size calculations in these reviews.

### Quality assessment

#### Internal validity/Risk of bias

The Cochrane Collaboration`s tool for assessing risk of bias will be used to assess the internal validity of the included studies [30]. This tool addresses sequence generation, allocation concealment, blinding of participants, personnel and outcome assessors, incomplete outcome data, selective outcome reporting, and other sources of bias. To assist the assessment standardized instructions have been developed based on the general instructions in the Cochrane handbook adapted for our purposes. These instructions will be updated as necessary (mainly if new, unpredicted issues come up). An overall assessment of bias will be performed by classifying all studies in the categories of low, unclear, and high risk of bias.

#### External validity

External validity (generalizability) will be addressed by documenting study setting, patient selection criteria, patient characteristics, clinical relevance of outcomes, length of follow-up, adverse effects, and discontinuation rates.

#### Procedures and consequences of quality assessment

Study quality will be assessed independently by two reviewers. Disagreements will be recorded and resolved by discussion.

If considerable methodological heterogeneity is present, subgroup analyses will be performed through comparing the findings between studies of low and unclear or high risk of bias.

### Synthesis of information from primary studies

#### Calculation of effect size estimate for single trials

For dichotomous effectiveness outcomes (response, remission, relapse) the ratio (with 95% confidence intervals) of responder proportions will be calculated (using both an intention to treat and a per protocol approach). The main outcome measure is response after completion of acute phase treatment. For continuous outcomes (depression scores) mean differences and standardized mean differences and their 95% confidence intervals will be calculated based on intention to treat data, available case data and per protocol data. As we assume that intention to treat data for continuous measures will not be available for a number of studies we will use a preference approach for collecting data for the primary meta-analysis (first preference intention to treat data, second preference available cases data, third preference per protocol data). For sensitivity analyses effect sizes will also be calculated assuming a fixed difference between the actual mean for the missing data and the mean assumed by the analysis [30]. For rare outcomes and safety outcomes (number of patients with adverse effects; discontinuation for any reasons, for lack of improvement or for adverse effect) odds ratios will be calculated.

#### Grouping experimental and control interventions for comparisons

If possible we will perform analyses on single pharmacological agents (e.g., citalopram) but we expect that the number of trials per agent performed in primary care will usually be very small. Therefore, we consider to categorize pharmacological interventions into five groups: 1) tricyclic and tetracyclic antidepressants; 2) selective serotonine reuptake inhibitors, 3) monoamino-oxidase inhibitors, 4) newer agents such as venlafaxine or mirtazepine, 5) hypericum extracts. If the literature search reveals further relevant groups the protocol will be amended. Typical comparator groups in the trials of these agents are either another pharmacological agent or placebo. The overwhelming majority of the trials of pharmacological trials are dealing with acute phase treatment and have durations up to 12 weeks.

Trials on psychological interventions are clinically highly heterogeneous. Existing meta-analyses [11,12] performed primary analyses on all types of interventions with subgroup analyses for cognitive behavioural therapy, problem solving therapy and other therapies. Comparator groups (usual care, waiting list, placebo, pharmacological intervention) and study duration are also highly variable. Without an in-depth analysis of the available studies we feel yet unable to predefine an exact grouping scheme. However, we plan to form groups of broadly similar psychological interventions and control interventions for the primary analysis instead of combining all highly heterogeneous interventions and comparators.

#### Calculation of pooled effect size estimates for direct comparisons

Meta-analyses (random effects model, inverse variance weighting) will be performed for direct comparisons of single interventions and groups of similar interventions and comparators (for example, SSRIs vs. tricyclics, SSRI vs. hypericum extracts, SSRIs vs. placebo etc.) and defined time-windows (up to 3 months and above). Primary analyses will be based on intention to treat data for dichotomous outcomes and on the preference approach described above for continuous data. We plan to use the random effects model rather than the fixed effects model, because we assume that the included studies will not be functionally equivalent and will show considerable clinical (concerning population, intervention) and methodological (design, quality etc.) heterogeneity. Statistical heterogeneity between study results will be tested for significance using Cochran's Q test and quantified using the I^2 ^statistic. Results will be visually displayed as forest plots. We will adjust for funnel plot asymmetry using a method by Rücker et al. [31,32].

#### Subgroup and sensitivity analyses

A priori defined subgroup analyses will be performed according to diagnosis (trials restricted to patients meeting criteria of major depression and other trials) and risk of bias (low vs. unclear/high risk). Sensitivity analyses will be performed using available cases and per protocol data. For continuous outcomes we will also perform a sensitivity analysis in which corrected means (see above) are used for trials with missing data which did not use an intention to treat analysis.

#### Meta-regression analyses

In case of considerable heterogeneity between study results in a specific comparison that cannot be explained by the defined subgroups, a series of a posteriori (explorative) meta-regression analyses will be performed to identify sources of heterogeneity. All meta-regression analyses will be performed using the restricted maximum likelihood estimate method.

#### Multiple treatment meta-analysis

If possible we aim to perform a multiple treatment meta-analysis in which several interventions will be compared simultaneously [33,34]. We expect that this will be possible for the pharmacological interventions reviewed as trials are likely to use similar methodology (comparators, duration, outcome measures). We are uncertain whether it will be possible to include psychological interventions as the information available to us suggests that these trials are very different from trials of pharmacological agents. However, comparing different psychological interventions could be an option. Due to these uncertainties all multiple treatment meta-analyses will be considered as exploratory. The analyses will be done within a Bayesian framework using WinBUGS code following Lu and Ades [35]. Results will be interpreted in the way described by Salanti et al. [36]. Before the analyses will be initiated details of the methods will be predefined in an analysis plan.

## Discussion

Practitioners do not only want to know whether there is evidence that a specific treatment is more effective than placebo, but also how the treatment options compare to each other. Therefore, we believe that a multiple treatment systematic review of primary-care based randomized controlled trials on the most important therapies against depression is timely. We assume that a formal multiple-treatment meta-analysis will be possible for comparing selective serotonine reuptake inhibitors (SSRIs), tricyclic antidepressants, and hypericum extracts. Our preliminary analysis of the literature suggests that it might be difficult to include psychological treatments into the formal meta-analytic comparison. Trials on psychological interventions typically use other control groups (usual care, waiting lists or not clearly defined pharmacological treatment) than trials on pharmacological interventions (which are usually placebo-controlled or compare two pharmacological treatments), tend to have longer duration and often use other measurement scales. While multiple treatment meta-analysis gains considerable popularity it is associated with considerable methodological problems and based on strong assumptions [27]. Therefore, our project will also be a case study to investigate whether multiple treatment meta-analysis can be applied to answer the important question which antidepressant treatment is most effective in the primary care setting.

## List of abbreviations

CGI: Clinical Global Impression Index; DSM IV: Diagnostic and Statistical Manual - American Psychiatric Association, Forth Revision; HAMD: Hamilton Rating Scale for Depression; ICD 10: International Classification of Disease - WHO, tenth revision; ICTRP: International Clinical Trials Registry Platform; MADRS: Montgomery-Asberg Depression Rating Scale; MeSH: medical subject heading; RCT: randomized controlled trial; SSRIs: selective serotonin reuptake inhibitors; WHO: World Health Organization.

## Competing interests

The authors declare that they have no competing interests.

## Authors' contributions

KL and AS had the study idea, KL sketched the research design and drafted the manuscript. All authors participated in the further development of the study methods. All authors read and approved the final manuscript.

## Pre-publication history

The pre-publication history for this paper can be accessed here:

http://www.biomedcentral.com/1471-2296/12/127/prepub
